# Congenital simple hamartoma of the retinal pigment epithelium with depigmentation at the margin in an Indian female

**DOI:** 10.3205/oc000112

**Published:** 2019-06-18

**Authors:** Koushik Tripathy, Gopal Bandyopadhyay, Koushik Basu, Kishore Kumar Vatwani, Himanshu Shekhar

**Affiliations:** 1 Department of Ophthalmology, ASG Eye Hospital, Kolkata, India

**Keywords:** congenital hypertrophy of the retinal pigment epithelium, CHRPE, Congenital simple hamartoma of the retinal pigment epithelium, CSHRPE, combined hamartoma of retinal and retinal pigment epithelium, CHRRPE

## Abstract

**Objective:** To report a case of congenital simple hamartoma of the retinal pigment epithelium (CSHRPE) with depigmentation at the margin.

**Method:** Case report.

**Result:** A 40-year-old Indian female was noted to have a small pigmented lesion with a depigmented margin toward the fovea in the right eye. The best-corrected vision was 6/6 in either eye. The optical coherence tomography revealed a highly reflective lesion at the retinal surface causing a shadow effect, suggestive of CSHRPE.

**Conclusion:** Imaging features of a patient with CSHRPE with a crescent-like depigmented area at the margin are presented.

## Introduction

Congenital simple hamartoma of the retinal pigment epithelium (CSHRPE) is a rare benign pigmented lesion of the ocular fundus. A PubMed search on 21^st^ October 2018 with the search phrases “simple hamartoma of retinal pigment epithelium” and “congenital simple hamartoma of retinal pigment epithelium” resulted in 22 papers discussing 31 cases and 3 review articles. However, these search results did not include the earliest description of the entity in 2 cases by Laqua [1] and 10 cases by Gass [2]. Gass [2] divided these lesions into 3 types – superficial, intraretinal with preretinal extension, and preretinal extension with superficial vascularization. The authors present a case of CSHRPE with depigmentation at the margin.

## Case description

**Figure 1:**
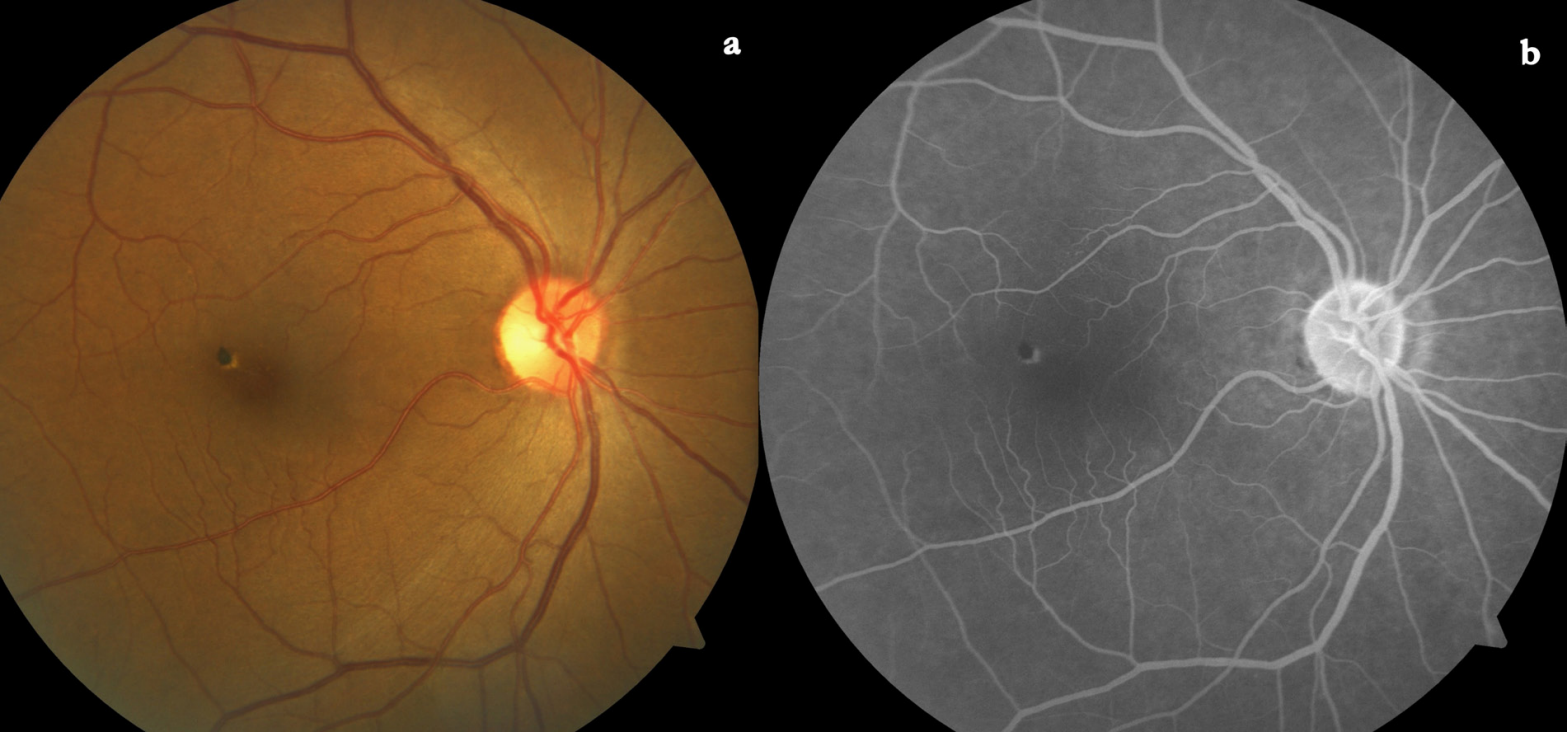
a) The fundus photo shows the sharply defined small pigmented lesion. b) The fluorescein angiogram showed block fluorescence at the area of pigmentation and window defect at the marginal depigmentation.

A 40-year-old Indian female presented to us for a routine check-up. The best corrected visual acuity was 6/6 and N6 in both eyes (+0.50/–2.00 x 90º in both eyes). The anterior segment was unremarkable in both eyes. Intraocular pressure was 12 mmHg in both eyes. The left fundus was normal. The right fundus showed a small pigmented flat lesion very near and superotemporal to the foveola. It had a crescent-like depigmented margin towards the foveola (Figure 1a (Fig. 1)). Fundus fluorescein angiogram (FFA) showed blocked fluorescence at the pigmented lesion and hyperfluorescence at the area of depigmentation (Figure 1b (Fig. 1)). 

**Figure 2:**
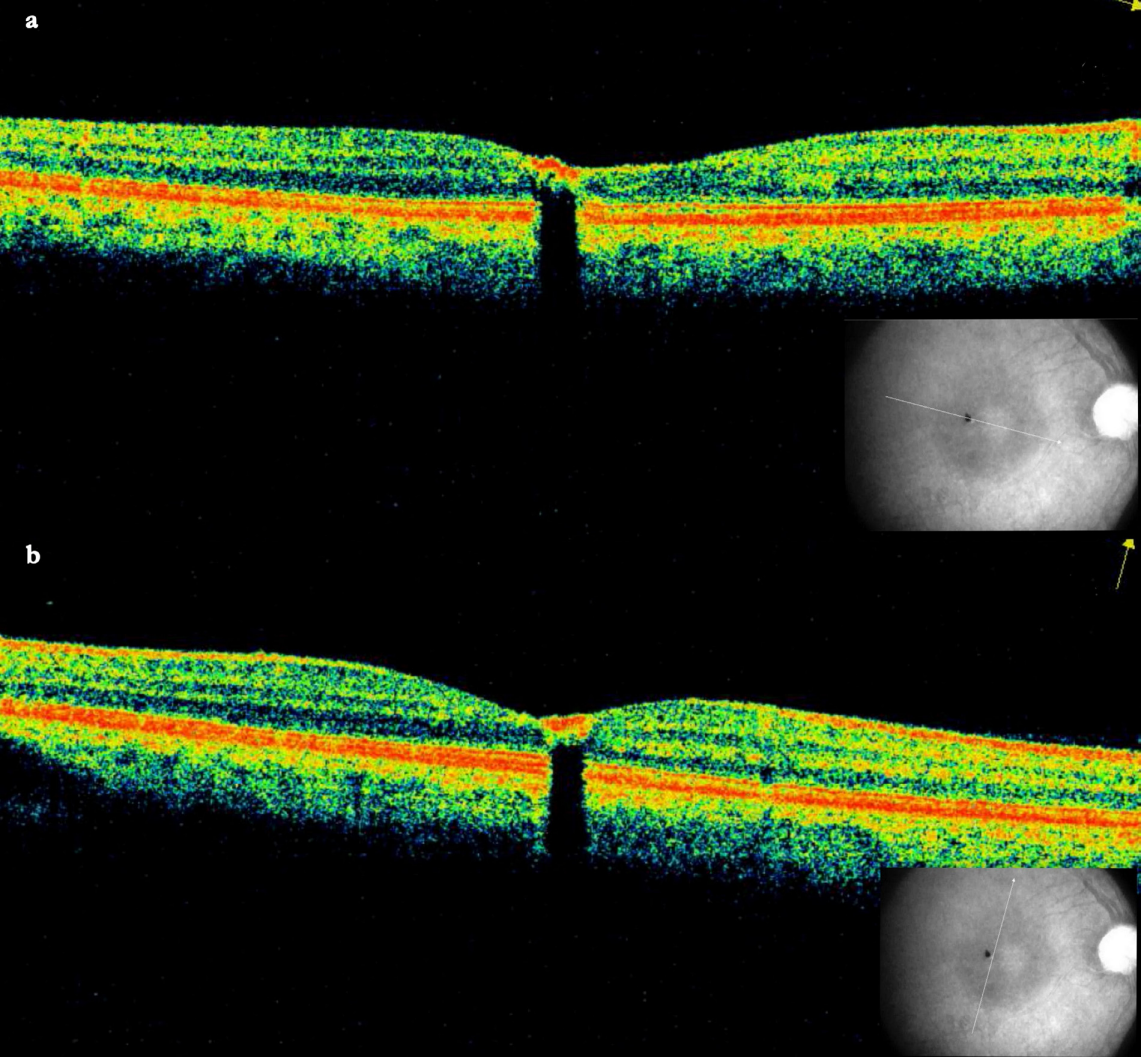
a) The OCT scan through the lesion, shows a hyperreflective lesion at the surface with shadow effect. b) The OCT scan through the depigmented margin shows surface lesion with shadow effect.

Optical coherence tomography (OCT) revealed a minimally elevated highly reflective lesion at the retinal surface causing a shadow effect, suggestive of CSHRPE (Figure 2a (Fig. 2)). The inner retinal hyperreflectivity was also present in the OCT scans through the depigmented area, suggesting it to be a part of the CSHRPE (Figure 2b (Fig. 2)).

## Discussion

Gass [2] defined the retinal pigment epithelial hamartoma as “focal, nodular, jet black lesions that usually appear to involve the full thickness of the retina and to spill onto the inner retinal surface in an umbrella fashion”. However, the lesions may be flat [2], [3], [4] or also minimally elevated [5], [6]. Other names of this lesion are congenital RPE adenoma, congenital or primary RPE hyperplasia [7], and RPE hamartoma [2]. The lesion is composed of hyperplastic RPE with variable vascular component [2]. The typical location is near the fovea, nasal peripapillary CSHRPE has also been reported [2]. These lesions typically do not cause visual decline or changes in the surrounding retina, retinal pigment epithelium (RPE) or choroid, or hemorrhage or exudates [2]. On long-term follow-up, decrease in vision may be seen. Other features include dragging of surrounding vessels for especially foveal lesion, vitreous adhesion/vitreomacular traction (VMT, which might gain some vision after vitrectomy and release of the VMT), mild retinal traction, minimally dilated retinal feeding artery and draining vein, retinal exudation, and pigmented vitreous cells [7], retinal hemorrhage, macular hole, congenital retinal arterial macrovessel, and intraretinal hyporeflective spaces (cystoid macular edema which partially responded to intravitreal bevacizumab). Reported associations include amblyopia, esotropia, visual decline, anomalous optic nerve head [2], and Coats’ disease like response.

The fluorescein angiogram shows hypofluorescence of the black lesion and hyperfluorescence at the depigmentation within the lesion [7] or at the margin with or without a hyperfluorescent halo [7]. Depigmentation within the lesion may correspond to the intrinsic vasculature of the hamartoma [8].

Marginal depigmentation has been reported in case 2 of the series by Baskaran and colleagues [3] and case 1 of the series by Kálmán and Tóth (this case had a history of trauma) [4]. In these cases, the depigmentation was at the margin toward the foveola. In our case, the depigmented area was on the inner surface of the retina rather than the RPE (Figure 2b (Fig. 2)) confirming it to be a part of the CSHRPE. A similar appearance may be due to focal RPE hyperplasia which may occur after various insults including trauma, intraocular inflammation, hemorrhage, and retinal detachment [9]. In particular, case 2 of the series reported by Shields and coworkers is similar to our case [9]. This male had a history of macular laser thrice for central serous chorioretinopathy. At 59 years of age, he developed “a focal area of flat pigmentation measuring approximately 200 mm in diameter [...] first noticed immediately temporal to the foveola, where the laser had been applied previously”. The lesion continued to grow gradually in diameter and thickness, and after 8 years “it was 2 mm in diameter and 2 mm in thickness and had circinate exudation around the elevated portion of the lesion with a rim of shallow subretinal fluid” [9]. The lesion continued to grow and eventually eroded through the retina and involved the posterior vitreous face [9]. In contrast to this patient, our patient did not have a history of ocular trauma or laser. Also, the fovea did not show any other abnormality than the pigmented lesion. Another differential of the lesion in our case may be congenital hypertrophy of the retinal pigment epithelium (CHRPE) which may have a depigmented halo around it. CHRPE involves the RPE in the OCT scan, in contrast to our case which was in the superficial retina. A combined hamartoma of the retina and the RPE [10] typically does not have a well-defined margin and is associated with epiretinal membrane/gliosis, vascular tortuosity/dragging, and deep pigmentation. In our case, the lesion was well-defined and there were no other associated changes. Macular telangiectasia type 2 was excluded in our case as the typical perifoveal graying, perifoveal leakage in FFA, and intraretinal tissue defects in OCT were absent. Other differential diagnoses were ruled out in our case which include a foreign body, adenoma or adenocarcinoma of the RPE, an intraocular foreign body and retinal invasion from an underlying choroidal tumor (nevus or melanoma) [7]. Ultrasound of the eye could not detect the lesion in our case. Autofluorescence and OCT angiogram were not done, and long-term follow-up or histological correlation was not available in our patient, which are limitations of this case report.

In conclusion, we describe a case of CSHRPE in an Indian female, who also had a depigmented crescentic area at the margin of the lesion.

## Notes

### Competing interests

The authors declare that they have no competing interests.
